# Continuous Compartment Pressure Monitoring Allows the Early Detection of Compartment Syndrome After Arterial Revascularization

**DOI:** 10.7759/cureus.55451

**Published:** 2024-03-03

**Authors:** Drew Schupbach, Rudy Reindl, Heather L Gill, A S Liberman, Edward J Harvey

**Affiliations:** 1 Surgery, McGill University, Montreal, CAN

**Keywords:** pain, sensors, compartment syndrome, trauma, vascular, surgery

## Abstract

Compartment syndrome (CS) occurs in several clinical scenarios. Reperfusion injury and tissue swelling are common causes. This can occur after trauma but also is seen post revascularization of extremities. CS is a difficult diagnosis to make in a timely fashion that avoids permanent tissue damage. The treatment for CS is immediate fasciotomy, but fasciotomy is not a complication-free procedure. Previous care pathways usually resulted in fasciotomy being performed in a disproportionate number of normal legs. These false positives and prophylactic releases are costly to the health system because of protracted hospital stays and increased surgery numbers. The desirable tool for surgeons would be one that decreases false positives and negatives while ensuring a diagnosis in a timely fashion with true positives. A new technology that allows continuous pressure monitoring seems to be the best aid to make a diagnosis. We present our experience in decreasing the time to diagnosis in a CS case post revascularization despite the neurological blockade.

## Introduction

Compartment syndrome (CS) is a limb-threatening condition. The diagnosis of acute CS still currently is thought to be clinical. It relies on clinical findings, and the findings are debated. The 6Ps of increasing pain, paresthesia, pallor, pulselessness, paralysis, and poikilothermia are in many references. Later thinking includes pain out of proportion to the injury, pain on the passive stretch of the muscles in the affected compartment, hypoaesthesia (and later anaesthesia), and weakness in the distribution of the nerves traversing the compartment, swelling, and tight compartments. Unfortunately, any clinical findings are subjective. Pressure measurement has been used to aid in the diagnosis by some investigators but with mixed results. Early methods and devices used for measuring intracompartmental pressure (ICP) have limited accuracy and reliability [1,2]. Monitoring continuous pressure has been shown to decrease the time to surgery and aids in the diagnosis [3]. Gold standard treatment of acute CS consists of an emergency fasciotomy aiming to immediately reduce the pressure within the compartment [4]. The surgical wound is often left open for several days, and the necessity for delayed closure of the wound carries a high risk of infection and delayed healing or potentially delayed healing and potential nonunion. These risks are not limited to therapeutic fasciotomies; in fact, prophylactic fasciotomies have shown similar or higher risks of complication [4-6]. Additionally, big data analyses have shown that almost one in six fasciotomies revealed some degree of myonecrosis, and 5.4% of fasciotomies led to amputations [7]. This indicates that current techniques used for the diagnosis of ACS may be inadequate, resulting in late treatment or unnecessary interventions. The use of a new micro-electrical machine systems (MEMS)-based pressure monitor [2] has shown good results in trauma patients [8].

## Case presentation

A 28-year-old male underwent pelvic exenteration surgery for a locally advanced stage of rectal cancer. He had a resection of rectal cancer 18 months previously. He had received neoadjuvant chemoradiotherapy at that time. He later underwent a planned ileostomy takedown and right lateral pelvic node dissection (LPND) six months thereafter. Unfortunately, he developed a significant local recurrence of his disease. He was offered radical local resection and was taken to the operating room for exenteration. This survey included resection of the colonic conduit and the previous anastomosis, as well as the right pelvic sidewall, seminal vesicle, and distal ureter. The surgery was complicated intraoperatively by an external Iliac artery injury. The injury was to the right external iliac artery and involved over 50% of the circumference. It was repaired with interposition of the internal iliac artery, which had previously been ligated as part of the exenteration, in order to avoid synthetic material in an infected field. Just over 3 cm of length was replaced, and a distal embolectomy was performed by the vascular surgery team. The complete duration of the exenteration and vascular surgery was 11 hours, performed in the Lloyd Davies position. This resulted in an avascular period of the right leg of at least four hours. The estimated blood loss for the surgery was 3 L. Resuscitation included five units of packed cells, two units of albumin and fresh frozen plasma, and 6,500 ML of crystalloid. The patient was taken to the post-anaesthesia care unit. An epidural was in place, which provided pain control. The orthopaedic care team was consulted three hours post surgery to rule out compartment syndrome.

The patient did not complain of excruciating pain. Examination of the right leg revealed normal colour (no pallor) and normal temperature (no poikilothermia). Pulses were equal bilaterally with good tissue oxygenation as per the foot-based oxygen sensor to the right foot. There were no paresthesias. The patient experienced some mild pain (3/10) in the right calf but no increase in pain with passive motion. By clinical exam, he did not have compartment syndrome (CS) [9]. According to the vascular team, he had experienced an avascular time not normally associated with reperfusion injury or a CS. He was judged to be at mild risk but also thought to be difficult to follow with serial clinical examinations alone [7,9,10]. It was opted to add continuous intracompartmental pressure monitoring in order to aid in the clinical examination.

**Figure 1:**
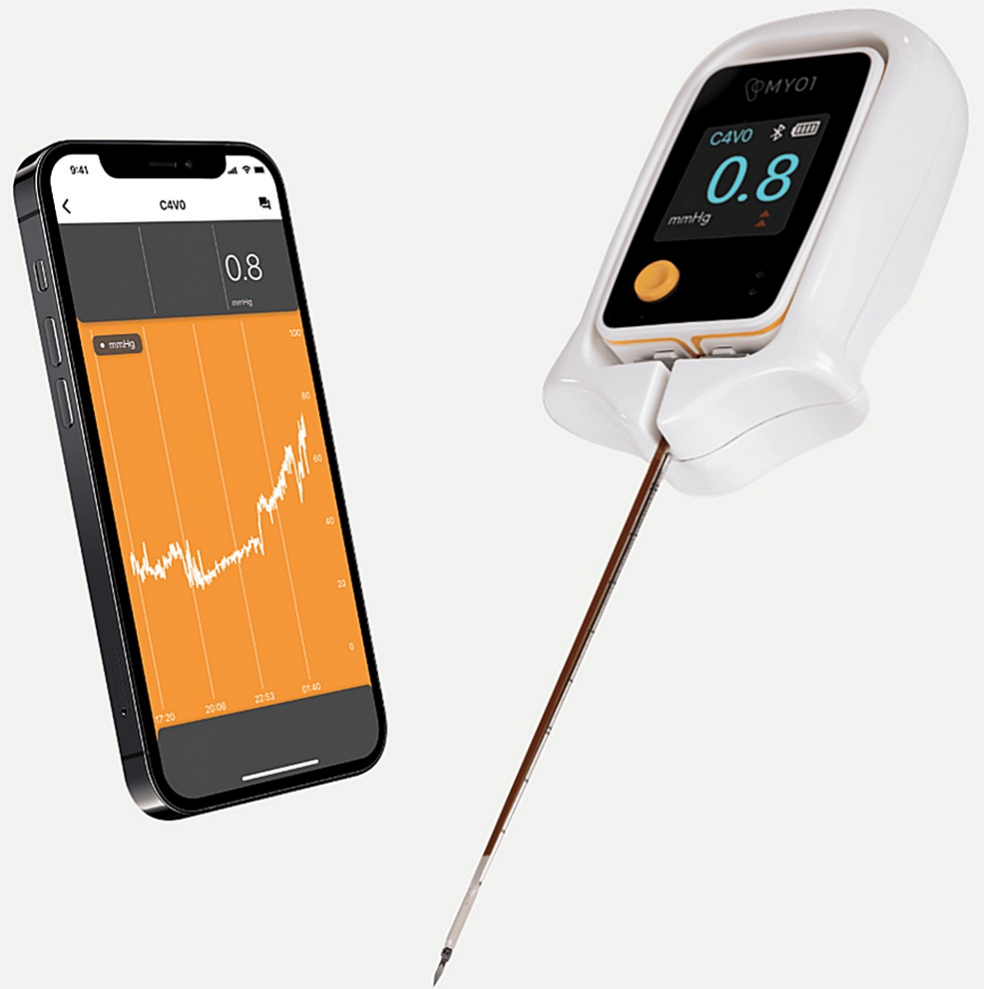
MEMS-based pressure monitor Commercially available FDA-approved device used for continuous compartment pressure monitoring. The sensor tip is placed in the muscle belly, and the trochar is then detached from the sensor, allowing the sensor to remain in place.

The MEMS-based pressure monitor [2] was inserted as per protocol in the anterior compartment of the right leg [11]. The image (Figure 1) was supplied by the manufacturer, MY01 Inc. (Quebec, Canada).

**Figure 2:**
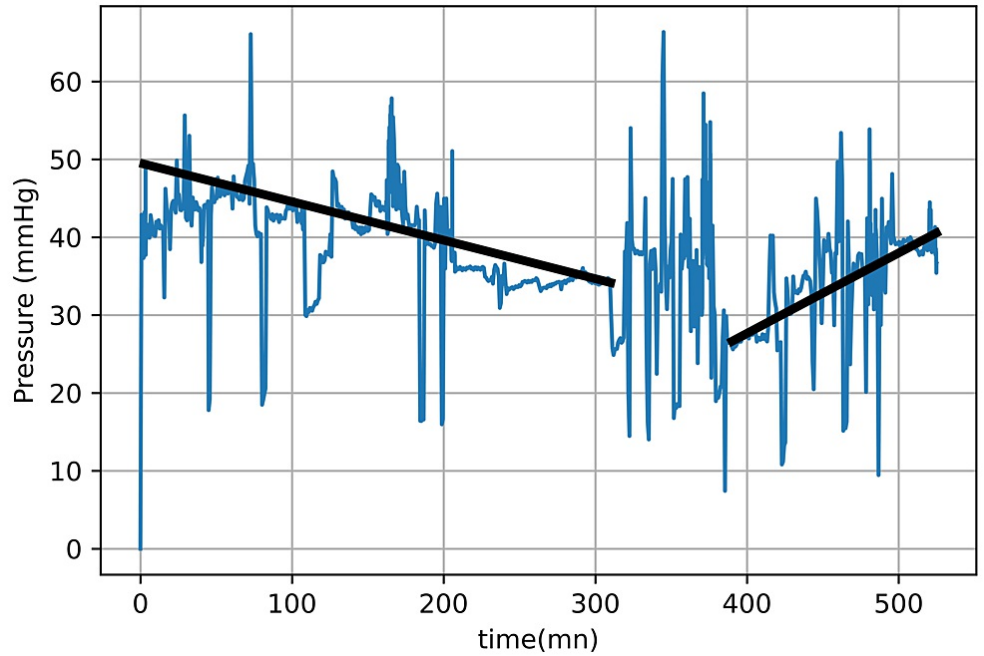
Intracompartmental pressure tracing from the anterior leg compartment Tracing obtained from the device app showing 530 minutes of data. The line on the left shows decreasing pressure for 300 minutes after device insertion. There is then a period of approximately 60 minutes where the tracing shows flat readings before the pressure starts to climb. The line on the right follows that climb in pressure. This is typical of the pressure tracings seen in reperfusion injuries before compartment syndrome develops.

Over the next six hours, the physical examination was unchanged. Figure 2 shows the pressure measurements over the night and the next morning. 

Pressure values trended downward as well for the first five hours. Thereafter, there was an inflection point in the pressure values where the absolute pressure was steady at 35 mmHg. The physical exam was unchanged, and there was no change in the presentation of the 6Ps. Following this point, there was then a three-hour (from six hours postoperative to nine hours postoperative) upward drift in the pressure values (Figure 2, line on the right of the figure), and, eventually, the delta P (diastolic minus ICP) was variably close to the values recommended by Leighton et al. [8] and McQueen et al. [10] for fasciotomy. At this time, nine hours after consultation, the patient began having more pain in his leg. Motion did not increase the pain generally, but there was more pain in the posterior compartment notably with ankle dorsiflexion. Blood pressure was 110/75-80 with a pulse rate of 70-90 at the time of the decision. Despite this confusing clinical presentation, it was elected at this time to take the patient to the operating room for four-compartment fasciotomies as it was felt that there may be an impending CS. A four-compartment two-incision release was accomplished with a full-length fasciotomy. At surgical treatment, this was found to be a Grade 2 CS. This is a true positive early case with bulging of the musculature such that primary closure was impossible [12]. The lateral compartment was the only muscle that was seemingly compromised with decreased circulation represented by a dusky color of the muscle that completely recovered during the surgical operation. The muscle was responsive to electrical stimulation. Early detection avoided permanent muscle death. Closure of the skin was accomplished at the next surgery 72 hours later.

## Discussion

As has been shown for the use of continuous pressure monitoring in trauma patients, this case of revascularization allowed early diagnosis before permanent muscle damage occurred. It has been difficult in the past to appropriately diagnose impending CS [9]. It has been seen in large database studies [7] that a disproportionate number of fasciotomies are being done to avoid missing the diagnosis. This has been seen with a high number of false negatives from reliance on only clinical exams [11]. An even higher number of false positives has resulted in a large number of unnecessary surgeries as well [11].

This device produces a graphic output sent wirelessly to a phone app, as well as a discrete pressure output on a display screen on the leg itself for direct pressure observation. The device allows pressure trend measurement and the calculation of perfusion pressure. MEMS-based sensors are a new technology with increased accuracy that allows the placement of a sensor directly into the muscle belly for a more physiological pressure value [2]. Preclinical and clinical studies have shown that this is more accurate than previous technology [8]. These same studies have also shown that only one compartment, ideally the anterior, needs to be monitored [8,11]. It also allows real-time pressure value monitoring in a continuous manner that has not previously been possible [2]. The literature around this new methodology has allowed the establishment of new standards in CS diagnosis and treatment. Trends upwards or downwards combined with clinical examination have shown high sensitivity and specificity for diagnosis. 

The important innovation in care with this method of continuous pressure monitoring is how it aids in the avoidance of unnecessary fasciotomies, and their resulting complications while facilitating early recognition of true positive findings. In orthopaedic trauma, this technique and technology has resulted in a reduction of OR time and unnecessary fasciotomies. Earlier work by McQueen et al. [10], amongst others, has been further validated with recent clinical data [8] on continuous compartment pressure monitoring. The trend over time is a newer concept that aids in CPM interpretation.

This patient was judged to be not immediately at risk for CS. The patient endured a short period of avascular injury relative to what is deemed acceptable in the literature. He did not have any clinical signs of CS even several hours after revascularization. Performing an immediate fasciotomy was not desirable for the many reasons discussed. We now know from the literature that clinical signs are not reliable [9] and that older techniques for pressure monitoring are unreliable [1,2,9]. Newer technology allows better aid in diagnosing CS, particularly for these patients.

## Conclusions

MEMS-based continuous compartment pressure measurement represents a new significant aid in the diagnosis of CS. Its use in preclinical and clinical traumatic cases has revealed high specificity and sensitivity, allowing early diagnosis and avoidance of false positives. Continuous compartment pressure monitoring has been shown, with data now reproducible over two decades, in order to decrease the time to the operating room in CS patients - where delaying surgery means tissue death. The new MEMS-based device allows remote pressure measurements to be made inside the muscle belly itself. This device was used in this patient and allowed early diagnosis of CS in this post-reperfusion case despite the neurological blockade. Continuous pressure measurement will play an important role in the diagnosis of true positive cases while decreasing the number of unnecessary surgeries (false positives and prophylactic surgeries). This represents a new method of controlling patient morbidity and minimizing hospital costs, as well as ensuring maximal patient outcomes.
